# Triptolide and its prodrug minnelide suppress Hsp70 and inhibit *in vivo* growth in a xenograft model of mesothelioma

**DOI:** 10.18632/genesandcancer.55

**Published:** 2015-03

**Authors:** Blake A. Jacobson, Esther Z. Chen, Shaogeng Tang, Holly Sedgwick Belgum, Joel A. McCauley, Kristen A. Evenson, Ryan G. Etchison, Joe Jay-Dixon, Manish R. Patel, Ahmad Raza, Ashok K. Saluja, Jonathan D'Cunha, Robert A. Kratzke

**Affiliations:** ^1^ Departments of Medicine, University of Minnesota, Minneapolis, Minnesota, USA; ^2^ Departments of Surgery, University of Minnesota, Minneapolis, Minnesota, USA

**Keywords:** Triptolide, minnelide, Hsp70, mesothelioma

## Abstract

Malignant mesothelioma is a devastating disease with a poor prognosis for which there is a clear need for more successful therapeutic approaches. Triptolide, a diterpenoid triepoxide, is a highly effective agent against several cancer types in animal models. Owing to triptolide's poor solubility in water, a water-soluble analog, minnelide, was synthesized. Minnelide is a prodrug of triptolide and is activated by exposure to phosphatases that are found in all body tissues, including blood. Mesothelioma cells were treated *in vitro* with minnelide or its parent compound, triptolide. Minnelide and triptolide were both found to significantly reduce mesothelioma cell viability and induce apoptosis. The level of Hsp70, a protein that promotes cancer cell survival, was measured in mesothelioma cells before and after treatment with triptolide. Hsp70 levels were decreased in a dose-dependent manner. In addition, triptolide sensitized cells to gemcitabine and pemetrexed as measured by cell viability. Mice bearing mesothelioma flank tumors were treated with daily injections (28 d) of minnelide or saline solution and xenograft tumor growth recorded. Mice displayed significantly reduced tumor burden. These findings support the clinical evaluation of minnelide therapy for mesothelioma.

## INTRODUCTION

Malignant mesothelioma (MM) is an aggressive malignancy related to asbestos exposure (1). The disease, which is responsible for the deaths of 3000 Americans annually (2), has a lengthy latency phase, sometimes 20 to 30 years subsequent to exposure. For the majority of patients afflicted with mesothelioma, systemic therapy remains the only choice of treatment. In terms of single-agent therapy, a wide variety of compounds from many different drug families have been tested against MM with overall response rates ranging from 10-30%. Currently, the standard of care for MM is combination therapy with cisplatin and pemetrexed. This therapeutic combination leads to an overall response rate of 41% (3), median time to progression of 7 months, and overall survival of 12 months (4). There is no recognized standard of care for second-line therapy. Therefore, it is important to develop new therapeutic targets and interventions to treat patients with this deadly disease.

*Tripterygium wilfordii* is a Chinese medicinal herb that has been used for centuries to treat inflammatory and autoimmune diseases (5-7). Triptolide, a diterpenoid triepoxide, is one of more than 100 components that have been isolated from *T. wilfordii*. The first report of triptolide's immunosuppressive and anticancer properties appeared in 1972 (8). Since then, triptolide has been shown to inhibit proliferation and induce apoptosis of many cancer types *in vitro* and prevent tumor growth *in vivo* (5, 9-11). The precise mechanism of how triptolide kills cancer cells is unknown. However, recent studies have shown that triptolide mediates cancer cell death by inhibiting expression of a member of the heat shock protein family, Hsp70 (heat shock protein 70) (11). Hsp70 is aberrantly expressed in several human malignancies and its inhibition kills cancer cells (12). In addition, Hsp70 expression is elevated in both pancreatic cancer and osteosarcoma compared to normal cells, and treatement with triptolide or minnelide, a water-soluble prodrug of triptolide, decreases Hsp70 expression and induces apoptosis and cell death in preclinical studies (11, 13). Furthermore, triptolide reduces Hsp70 expression and inhibits cancer cell proliferation in neuroblastoma (11, 14). Elevated levels of Hsp70 are also implicated in increased resistance of MM cells to chemotherapeutic drugs (15-17). These studies suggest that Hsp70 may be a potentially important therapeutic target for mesothelioma.

The use of triptolide in animal models has been restricted because of its poor solubility in aqueous medium (18). Therefore, a water-soluble prodrug of triptolide named minnelide was developed. To date, investigations have demonstrated that minnelide inhibits growth of xenografts of non-small cell lung cancer (19), osteosarcoma (13), and pancreatic cancer (18). In this study, minnelide was assessed as a therapeutic agent against mesothelioma. Mesothelioma cell viability was reduced and apoptosis induced by minnelide and triptolide. Triptolide treatment sensitized cells to pemetrexed and gemcitabine. Triptolide exposure decreased cellular levels of Hsp70 in a dose-dependent manner. Importantly, intraperitoneal delivery of minnelide into mice bearing MM xenografts significantly suppressed tumor growth. These preclinical studies support the clinical development of minnelide as a novel agent for mesothelioma therapy.

## RESULTS

### Triptolide and the prodrug minnelide repress mesothelioma proliferation

**Figure 1:**
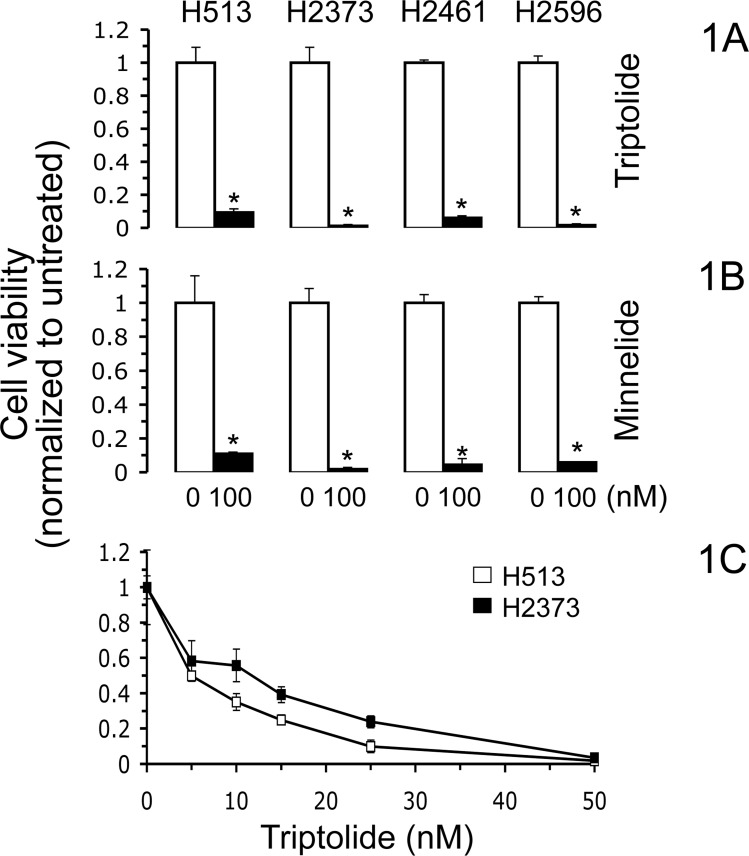
Triptolide or minnelide treatment suppresses mesothelioma proliferation Mesothelioma cell lines H513, H2373, H2461, and H2596 were untreated or treated with 100 nM of triptolide (A) or minnelide (B). For dose response analysis, H513 and H2373 cell lines were untreated or treated with the indicated concentrations of triptolide. Viable cells were counted 72 hours after treatment. For minnelide treatment, antarctic phosphatase (2 units/mL) was present in the medium for the bioconversion of minnelide to the active compound triptolide (B). DMSO (0.2%) was present in all treatments containing triptolide (A, C). Data represent the mean (± SD) of three independent determinations of cell number normalized to untreated cells (A, B). Asterisks indicate a significant difference in cell viability (*p* < 0.05) between treated and untreated cells.

Previous research revealed that triptolide and its prodrug minnelide inhibited proliferation in a wide variety of cancer types (5, 9-11, 13, 18, 19). To investigate whether triptolide and minnelide inhibit proliferation in mesothelioma, we treated four MM cell lines with both drugs and assessed cell survival. At a concentration of 100 nM, both drugs significantly reduced cell viability to less than 11% of untreated control (Fig. 1A, B). The prodrug minnelide was roughly as potent as the active compound triptolide. Two different mesothelioma subtypes were included in this study, epitheliod (H2461) and sarcomatoid (H2373 and H2596), revealing that both minnelide and triptolide are equally effective against both mesothelioma subtypes. To elucidate the IC _50_ of triptolide in MM cells, the least (H513) and most (H2373) sensitive cell lines were treated with a range of drug concentrations. The IC _50_ for H513 and H2373 was 6.28 and 4.24 nM, respectively. These values are consistent with the values determined for lung cancer (19), osteosarcoma (13), and pancreatic cancer (18).

### Triptolide treatment diminishes Hsp70 protein levels

**Figure 2:**
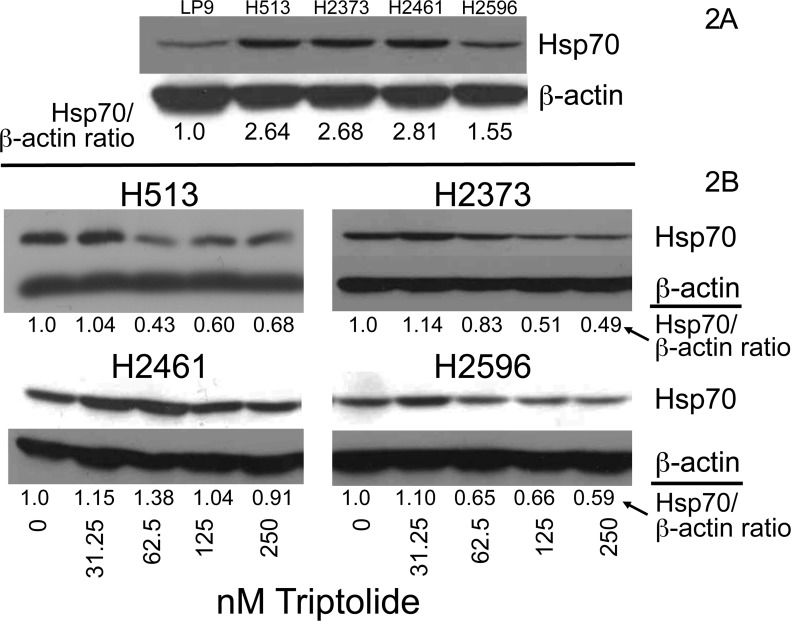
Hsp70 expression is reduced by triptolide in mesothelioma A. Immunoblot analysis of steady-state levels of Hsp70 in cultures of mesothlioma cell lines and normal mesothelial LP9 cells. The normalized ratio of Hsp70 to β-actin in LP9 cells compared with mesothelioma cell lines is shown at bottom. B. Cultured mesothelioma cells were treated with increasing doses of triptolide, and Hsp70 and β-actin protein expression was evaluated by immunoblot analysis from lysates harvested after 48 hours. The normalized ratios of Hsp70 to β-actin after triptolide treatment in mesothelioma cells is shown below each panel. ImageJ, a public domain java image processing program, was employed to measure protein band density.

Triptolide has been shown to reduce Hsp70 expression in pancreatic cancer and neuroblastoma (11, 14). To determine whether the malignant phenotype of mesothelioma is in part driven by overexpression of Hsp70, we examined steady-state levels of Hsp70. In a panel of MM cell lines, protein band density measurement following immunoblot analysis revealed that expression of Hsp70 was substantially increased compared to non-transformed human mesothelial cells (LP9) (Figure 2A). The mean increase for the MM cell lines was 2.4 fold (range 1.55-2.81). We next assessed whether triptolide exposure affects Hsp70 expression. MM cells were treated with increasing doses of triptolide for 48 hours and lysates prepared (Figure 2B). Three of the four MM cell lines exhibited decreased Hsp70 protein levels in a subtypes. To elucidate the IC of triptolide in MM cells, dose-dependent fashion. There was a modest increase in Hsp70 at the lowest dose used (31.24 nM). This initial increase in Hsp70 level after low-dose triptolide treatment may be a stress response of drug treatment as has been seen previously in mesothelioma (15, 16) and other cancers (20). H2461 cells displayed a boost in Hsp70 at triptolide concentrations of 31.25 and 62.5 nM followed by a reduction in levels at the highest doses. In addition, baseline Hsp70 levels were greatest for H2461 cells compared to other MM cell lines (Figure 2A). Together, these data show that at higher triptolide doses Hsp70 expression decreases substantially, in three of four MM cell lines, compared to untreated and low-dose treatment.

### Apoptosis is induced by triptolide and minnelide in mesothelioma

**Figure 3:**
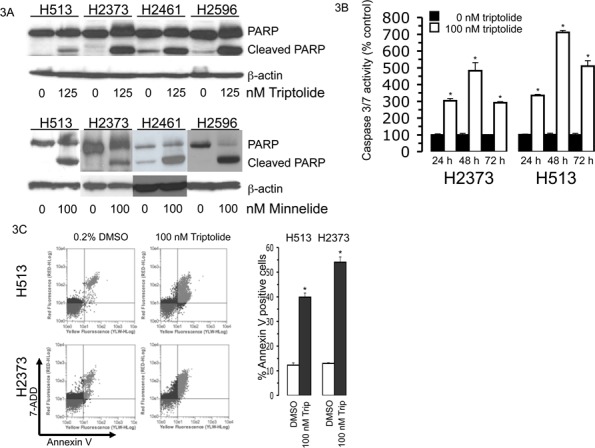
Triptolide or minnelide treatment induces apoptosis in mesothelioma A. Triptolide treatment induces PARP cleavage in mesothelioma. Mesothelioma cell lines were treated with the indicated concentrations of triptolide or minnelide for 48 hours. Lysates were immunoblotted with anti-PARP antibody. β-actin served as a loading control. B. Caspase 3/7 is activated after triptolide exposure. Mesothelioma cells were exposed to 100 nM triptolide for 24, 48, or 72 hours, and Caspase-3 and -7 activities were assessed. DMSO (0.2%) was present in all treatments. *Columns*, the mean of three independent determinations of caspase 3/7 activity normalized to untreated cells, *bars*, s.d. Asterisks denotes a significant difference in caspase 3/7 activity (*p* < 0.05) between treated and untreated cells. C. Annexin V staining is elevated in triptolide-treated mesothelioma cells. Flow cytometry analysis results of H513 and H2373 cell lines treated with triptolide or vehicle (0.2% DMSO) (left panel). Graphic depiction of the percent annexin V stained cells following treatment (right panel). Results are expressed as the mean of three independent determinations, *bars*, s.d. Asterisks indicate a significant difference in annexin V staining (*p* < 0.05) between treated and untreated cells.

Hsp70 upregulation can confer resistance to apoptosis (12, 20). We explored the possibility that suppression of Hsp70 levels by triptolide and minnelide treatment would lead to apoptotic cell death in mesothelioma. Several markers of apoptosis were investigated including poly (ADP-ribose) polymerase (PARP) cleavage, Annexin V staining, and caspase activity. PARP cleavage was assessed in MM cells treated with triptolide or minnelide as an indicator of induction of apoptosis. A panel of 4 MM cell lines were either treated with triptolide (125 nM) or minnelide (100 nM) or left untreated for 48 hours and lysates prepared. Immunoblot analysis revealed treatment with both triptolide and minnelide led to increased PARP cleavage in MM cells signifying apoptosis as compared to untreated cells (Figure 3A). Two cell lines were assayed for caspase 3/7 activity, which is an earlier indicator of apoptosis than PARP cleavage. H513 and H2373 exposed to 100 nM triptolide for 24, 48, and 72 hours resulted in a significant increase in caspase 3/7 activity compared to untreated cells, further linking reduced cell viability with activation of apoptosis (Figure 3B). The externalization of phosphatidylserine is a consequence of apoptosis that can be measured by Annexin V staining. MM cell lines H513 and H2373 were treated with vehicle (0.2% DMSO) or 100 nM triptolide for 48 hours, and cells were then subjected to Annexin V measurement. The level of Annexin V-positive cells increased from 12.3 to 39.8% for H513 cells and from 13 to 54% for H2373 cells (Figure 3C). On the basis of the results from these three different apoptosis assays, triptolide and minnelide are capable of inducing apoptotic cell death in mesothelioma cells.

### Triptolide treatment enhances susceptibility of mesothelioma cells to cytotoxic drugs

**Figure 4:**
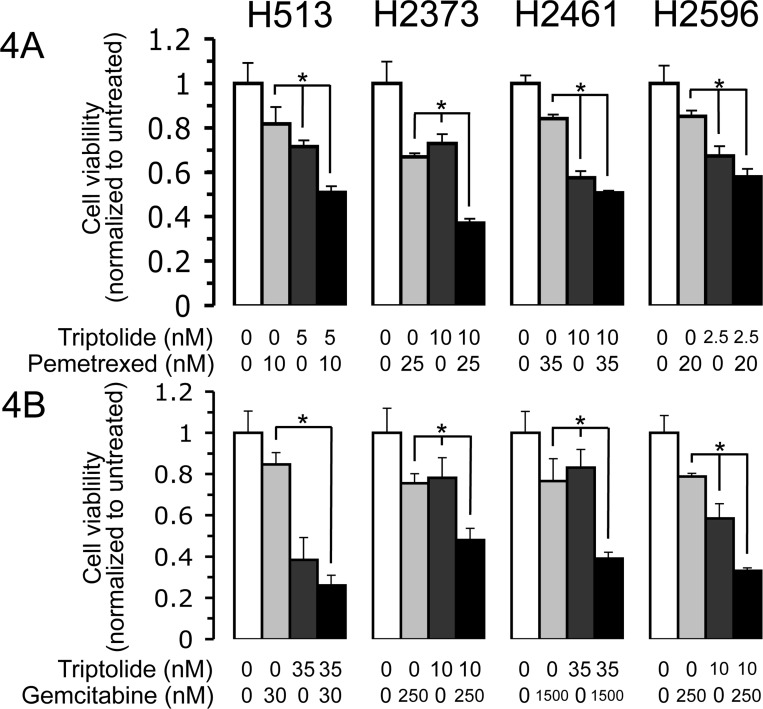
Triptolide enhances susceptibility of mesothelioma cells to cytotoxic drugs Mesothelioma cell lines treated with triptolide were treated with the indicated concentrations of gemcitabine or pemetrexed. Viable cells were counted after 72 hours. DMSO (0.2%) was present in all treatments. *Columns*, the mean of three independent determinations of cell number normalized to untreated cells, *bars*, s.d. Averages of combination treatment were compared to either agent alone by Student's *t*-test. Asterisk signifies a *p* value < 0.05.

To explore the possibility that triptolide treatment would also enhance chemotherapy-induced cell killing, we treated mesothelioma cell lines with pemetrexed and gemcitabine alone and in combination with triptolide. Cell viability was reduced among all four cells lines treated for 72 hours with the combination of triptolide and pemetrexed compared to each single agent treatment (Figure 4A). In each case, reduced cell viability for the combined treatment was significantly different than that of each drug alone. However, for H2461 the difference between triptolide alone and the combined treatment was not that extensive. The addition of triptolide treatment enhanced pemetrexed-induced cell death by 30.8% [H513], 29.8% [H2373], 33.6%, [H2461], and 27.2% [H2596] as compared to cells treated with only pemetrexed. Combination treatment with triptolide and gemcitabine also resulted in additional cytotoxicity compared to either treatment alone (Figure 4B). In three of the 4 cell lines studied, combined treatment resulted in cell viability that was significantly lower than either drug alone. Further, the addition of triptolide treatment increased gemcitabine-induced cell death by 58.7% [H513], 27.6% [H2373], 37.7%, [H2461], and 45.7% [H2596] as compared to gemcitabine alone.

**Figure 5:**
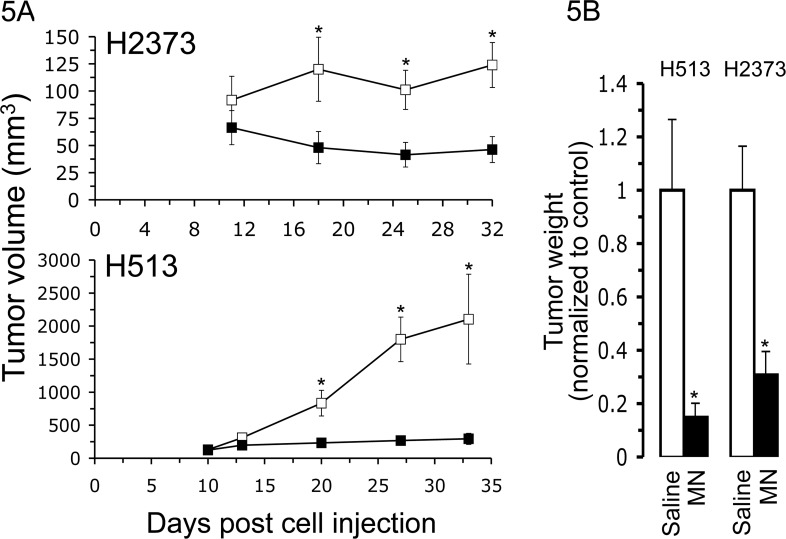
Minnelide potently inhibits xenograft tumor growth A. Tumor volumes were compared between minnelide treated (*n* = 10) and untreated groups (*n* = 10) in two xenograft models of human mesothelioma, H2373 and H513. Mice began receiving daily intraperitoneal injections of minnelide at 0.42 mg/kg mouse weight five days after tumor cell injection. Equivalent volumes of phosphate-buffered saline were injected into control mice. Extensive suppression of tumor growth occurred in minnelide-treated groups compared to control groups. B. Tumors were excised and weighed. Results are normalized to the untreated group for each cell line and expressed as the mean, *Columns*, *bars*, s.d. Asterisks indicate a significant difference in tumor volume and tumor weight (*p* < 0.05) between treated and control groups.

### Minnelide potently inhibits xenograft tumor growth

The promising *in vitro* results presented in Figure 1 demonstrating potent sensitivity of mesothelioma cells to minnelide along with the successful preclinical *in vivo* minnelide investigations (13, 18, 19) led to experiments to test the activity of minnelide against human MM in nude mouse xenografts. MM cells were implanted in the flanks of nude mice, and five days later mice were treated with either minnelide (0.42 mg/kg mouse weight) or PBS for twenty-eight days. Mice were sacrificed the 32nd (H2373) or 33rd (H513) day after tumor cell injection. Intraperitoneal injections of minnelide produced substantial activity against both H513 and H2373 tumors, resulting in markedly decreased tumor progression compared to PBS-treated controls. Significantly reduced tumor volume (p<0.02) at the completion of the experiment was 3.25 fold for H2373 and 7.1 fold for H513. The final weight of tumors from minnelide-treated xenografts was 31% (H2373) and 14.8% (H513) of the tumor weight of PBS-treated mice, respectively (p<0.006). Tumor volumes were found to be significantly different (p<0.05) starting on day 18 and day 20 after tumor implantation for H2373 and H513, respectively. Overall, these results show that when mesothelioma xenografts are treated with minnelide, tumor growth is drastically inhibited.

## DISCUSSION

The present study demonstrates that triptolide or minnelide treatment suppresses mesothelioma proliferation in part through suppression of Hsp70 expression and induction of apoptosis. Triptolide also potentiated cell killing when combined with cytotoxic drugs. Importantly, our data demonstrate that minnelide potently inhibits xenograft tumor growth. These data suggest that minnelide could potentially be an effective therapy for patients with mesothelioma.

Most human malignancies exhibit increased levels of Hsp70 family members, and overexpression of Hsp70 family members can indicate poor prognosis (12, 20). Enhanced Hsp70 levels protect cancer cells from apoptosis and cellular pressures linked to amplified growth, aneuploidy, and accumulation of mutant proteins (20). Abnormal levels of Hsp70 have been implicated in T-cell lymphoma in transgenic mice (21), breast cancer cell growth (22, 23), and tumorigenicity of Rat-1 fibroblasts (24). These studies indicate that Hsp70 is an attractive target for therapeutic intervention. One of the means by which triptolide is believed to inhibit cancer cell growth is by repression of Hsp70 expression. Triptolide exposure has been shown to lead to a decrease in Hsp70 expression in pancreatic cancer (11), osteosarcoma (13), neuroblastoma (14), and, in this study, mesothelioma. A recent study has revealed that the likely mechanism for the decreased levels of Hsp70 is that triptolide induces the upregulation of a microRNA, miR-142-3p, which directly binds to the 3′ UTR of *Hsp70 mRNA* and causes the destruction of *Hsp70 mRNA* (25). These studies were done in pancreatic cancer, but it is likely that triptolide functions similarly in other cancers. In the same study, minnelide was also shown to induce miR-142-3p levels in mice bearing human pancreatic tumors while concurrently reducing *Hsp70 mRNA*. It is probable that both triptolide and minnelide treatment of mesothelioma cells stimulated miR-142-3p, thus reducing aberrantly high Hsp70 levels and preventing cell growth as evident in the studies presented here. In addition to miR-142-3p, there are many other potential targets of triptolide or minnelide. Triptolide and minnelide resulted in robust cytotoxicity and induction of apoptosis in H2461 without substantial change in Hsp70 levels suggesting that Hsp70 does not play a major role in this cell line. DNA microarray investigations indicate that triptolide treatment upregulates 160 genes and downregulates 1511 genes in the non-small cell lung cancer cell line A549 (26). A recently identified target of triptolide is XPB (xeroderma pigmentosum type B), a subunit of transcription factor TFIIH (transcription factor II human). XPB becomes covalently bound when exposed to triptolide. This binding inhibits the DNAdependent ATPase activity of XPB and leads to inhibition of RNA polymerase II-mediated transcription. Inhibition of XPB may explain much of the biological activities of triptolide that result in decreased expression of target genes (27). Other potential target genes for triptolide in a variety of cancers include caspase 3,8 and 9; XIAP; bcrabl; Bax; Bcl-2; p53; p21; NFκB; MRP-1; ERK-1/2; JNK-1/2; p38 MAPK; PI3K; 5-LOX; ADAM10; jak2; mcl-1; and histone methyltransferase (5). This large number of cellular targets may also play a role in the antineoplastic efficacy of triptolide in some cell lines, such as H2461.

In conclusion, our data showing a significant reduction of tumor burden in mesothelioma xenografts support further clinical development of minnelide as a therapy for patients with mesothelioma. Furthermore, minnelide could be rationally combined with chemotherapy because Hsp70 is thought to be a chaperone of mediators of chemotherapy resistance (12). This approach is supported by our *in vitro* data suggesting that triptolide can be effectively combined with either gemcitabine or pemetrexed. The active compound triptolide was employed in these *in vitro* studies, but the water-soluble prodrug minnelide would be utilized in *in vivo* investigations. The standard of care for MM is combination therapy with cisplatin and pemetrexed. Our data support future testing of minnelide in combination with standard chemotherapy where it would be expected to deplete a cell of Hsp70, thus enhancing the activity of chemotherapeutic agents. The data presented here support the use of minnelide in combination with commonly prescribed first- and second-line chemotherapy regimens for mesothelioma. Improvement in the chemotherapy response would be expected to favorably impact outcomes for these patients. The safety of combinatorial approaches would have to be empirically proven in appropriately designed phase I studies. Alternatively, our data also suggest that minnelide alone has substantial antitumor activity in mesothelioma. Minnelide is currently in a phase I clinical trial for patients with advanced gastrointestinal (GI) tumors (clinical trial identifier number: NCT01927965). Once the maximum tolerated dose is determined and the established recommended dose of minnelide is obtained, a phase 2 study could be designed for treating patients with mesothelioma as a single agent.

## MATERIALS AND METHODS

### Cell lines and Cell culture

MM cell lines H513, H2373, H2461 and H2596 (American Tissue Culture Collection) were cultured in RPMI 1640 (Gibco, Invitrogen) containing 10% calf serum (Biofluids). LP9 cells, non-transformed human mesothelial cells, (National Institute on Aging Cell Repository), were maintained in a medium containing a 1:1 ratio of M199 and MCDB10 basal medium (Sigma) supplemented with 15% calf serum [not heat inactivated], 2mM glutamine, 10 ng/mL EGF and 0.4 μg/mL hydrocortisone. All cells were were maintained at 37oC in 5% CO_2_.

### Cell lysate preparation

Cells were removed from plates by scraping after washing once with PBS (phosphate buffered saline), after which cells were collected by centrifugation (14K rpm, 14 seconds), washed again with ice cold PBS followed by another round of centrifugation and resuspended in TNESV lysis buffer (50 mM Tris-HCl, pH 7.4; 1 % NP-40; 2 mM EDTA, pH 8.0; 0.1 M NaCl) containing protease (Roche) and phosphatase inhibitors (Sigma).

### Western blot analysis

Protein samples were separated by 10% SDS-PAGE (polyacrylamide gel electrophoresis) or by 8-15% gradient gels. After protein transfer to PVDF (polyvinylidene difluoride), the membranes were blocked in 5% non-fat dry milk for 1 hour at room temperature in Tris-buffered saline-Tween (TBST: 0.15 M NaCl; 0.01 M Tris-HCl, pH 7.6; 0.05% Tween 20). Membranes were then incubated for 1 hour at ambient temperature or overnight at 4o C with the chosen primary antibody. The primary antibodies employed were rabbit α-HSP70 antibody (Cell Signaling), rabbit α-PARP antibody (Cell Signaling), each at a 1:1000 dilution, and mouse α-β-actin (Sigma) at 1:10,000 dilution. Before and after incubation with the appropriate horseradish peroxidase labeled secondary antibodies, the blots were washed three times for 5 minutes in TBST. Detection was performed utilizing ECL Plus Western Blotting System (Amersham Biosciences) to visualize the bands of interest. Protein band density was measured by using ImageJ, a public domain java image processing program.

### Cell proliferation assays

Cells were seeded as triplicate sets into 6-well plates with 150,000 cells per well for H513, H2373, H2461, and H2596. After overnight incubation, cells were treated with 100 nM triptolide (Calbiochem, EMD Chemicals, Inc.) or minnelide. Control cells were treated with an identical concentration of vehicle in each experiment (0.2% DMSO for triptolide, 1 × PBS for minnelide). For the bioconversion of minnelide to the active compound triptolide, antarctic phosphatase (New England Biolabs, Inc.) (2 units/mL) was present in the medium during treatment. H513 and H2373, in a separate experiment, were treated as above except that varying doses of triptolide were used while keeping the concentration of vehicle identical for all cell treatments. Seventy-two hours later, cell number was determined by counting viable cells after exposure to trypan blue. Cell survival was normalized to vehicle-treated cells. Each experiment was performed in triplicate. The IC of triptolide for H513 and H2373 was determined by using Compusyn (ComboSyn, Inc., Paramus, NJ).

### Enhanced cytotoxicity assays

For treatment conditions that included pemetrexed, cells from each cell line were seeded (1.5 × 105 per well) in six-well plates. After overnight incubation, the medium was replaced with fresh medium or medium containing pemetrexed, triptolide, or pemetrexed plus triptolide at the indicated concentrations. Vehicle concentration was identical for all wells. Seventy-two hours later, cells were washed twice with PBS, trypsinized, collected, and resuspended. Cell number was then determined by counting viable cells after trypan blue treatment. Each experiment was done in triplicate. Three thousand cells of each cell line were seeded as triplicate sets onto 96-well plates for cytotoxicity assessment with gemcitabine HCL (Eli Lilly). After overnight incubation, cells were treated with the indicated concentrations of gemcitabine, triptolide, or both compounds. Control cells were treated with identical concentrations of vehicle. Cell viability was determined following seventy-two hour incubation using Cell Counting Kit-8 (Dojindo Molecular Technologies). 10 μL of CCK-8 solution was added to each well and plates incubated for 2 hours at 370C. Absorbance was measured at 450 nm. Cell survival was normalized to vehicle-treated cells. Experiments were performed in triplicate.

### Annexin V measurement

Phosphatidylserine externalization was determined as an indicator of apoptosis utilizing Guava Nexin Reagent and the Guava EasyCyte flow cytometer (Gauva Technologies). Cells were treated as above in six-well plates for 48 hours. The Guava Nexin reagent was added to harvested cells and incubated in the dark for 20 minutes at room temperature. Samples were analyzed using flow cytometry per the manufacture's recommendation. Results are expressed as the percentage of gated cells positive for Annexin V staining. Each sample was analyzed in triplicate.

### Caspase activity assessment

Caspase-3 and -7 activities were measured using the Caspase-Glo 3/7 assay kit (Promega) following the instructions of the manufacturer. 3000 cells were seeded into 96-well white-walled opaque plates and incubated overnight. Cells were then treated or not treated with 100 nM triptolide for 24, 48, or 72 hours, followed by addition of 100 μL of Caspase-Glo reagent to each well and 3 minutes of incubation at room temperature. Luminescence for each sample was measured using a plate-reading luminometer (FLUOstar Omega from BMG Labtech). Caspase activity was normalized to untreated cells. Experiments were performed in triplicate.

### Animal experiments

5×106 H2373 or 2.5×106 H513 cells in 0.1 mL 1X PBS were injected into the flanks of 4-6 week old nude mice (nu/nu; NCI) using a 25 gauge needle. Mice were sorted into treatment and control cohorts immediately after cell implantation. Five days after tumor injection, mice received daily intraperitoneal injections of minnelide 0.42 mg/kg at a concentration of 0.1 mg/mL. Corresponding volumes of phosphate-buffered saline were injected into control animals that would represent the volume of minnelide per dose. Tumor sizes were measured on the indicated days after tumor implantation. The long (D) and short diameters (d) were measured with a digital caliper (Fisher). Tumor volume (mm3) was determined as V = d2 × D × 0.5. Mice were sacrificed the 32nd (H2373) or 33rd (H513) day after tumor cell injection. Excised tumors were weighed. Each cohort consisted of ten mice. All procedures involving animals were performed according to guidelines of the Institutional Animal Care and Use Committee of the University of Minnesota.
